# mTOR Signaling in Cardiometabolic Disease, Cancer, and Aging 2018

**DOI:** 10.1155/2019/9692528

**Published:** 2019-02-04

**Authors:** Anindita Das, Flávio Reis, Paras Kumar Mishra

**Affiliations:** ^1^Pauley Heart Center, Virginia Commonwealth University, Richmond, Virginia 23298, USA; ^2^Institute of Pharmacology & Experimental Therapeutics, Coimbra Institute for Clinical and Biomedical Research (iCBR), Faculty of Medicine, University of Coimbra, 3000-548 Coimbra, Portugal; ^3^CNC.IBILI Research Consortium & CIBB Research Consortium, University of Coimbra, 3004-517 Coimbra, Portugal; ^4^Department of Cellular & Integrative Physiology, and Department of Anesthesiology, University of Nebraska Medical Center, Omaha, NE 68198, USA

The mammalian target of rapamycin (mTOR), an atypical multidomain serine/threonine kinase of the phosphoinositide 3-kinase (PI3K)-related kinase family, elicits a significant role in diverse signaling cascades responsive to changes in intracellular and environmental conditions. Activation of mTOR has been implicated in an increasing number of pathological conditions, including cancer, obesity and diabetes, cardiovascular diseases, and neurodegenerative disorders. Based on its pathophysiological importance, the mTOR signaling pathway has attracted unprecedented attention among basic scientists and clinicians. Growing empirical evidences demonstrate the pivotal role of mTOR signaling in oxidative stress, aging, proliferative disorders, and metabolic abnormalities. The current special issue is aimed at bringing together both original research papers (7 articles) and review articles (4 articles) to advance our understanding of mTOR signaling pathways in metabolic and cardiovascular diseases, cancer, muscle toxicity, and aging (Figure 1). Internationally recognized experts highlighted the distinct role of mTOR signaling in cardiovascular and metabolic diseases as well as cancer and neuronal tissue with insightful presentations to enrich our knowledge in emerging therapeutic application of mTOR inhibitors. Specific contributions to this special issue are summarized below.

In the previous special issue on “mTOR Signaling in Cardiometabolic Disease, Cancer, and Aging, 2017,” Dr. Pulakat's group reported the chronic treatment with rapamycin (Rap, a mTORC1 inhibitor) reduced the obesity and cardiac fibrosis in Zucker obese rats (ZO-C), while increasing their blood glucose levels. In contrast, rapamycin treatment induced cardiac fibrosis in heathy Zucker lean (ZL) rats, suggesting that mTORC1 inhibition exerts differential effects on diabetic versus healthy hearts. In the present study, A. M. Belenchia et al. demonstrated differential expression profiles of cardiac miRNAs between control and rapamycin-treated ZO and ZL rats to evaluate the mechanisms underlying adverse effects of rapamycin. They reported that 47% of rapamycin-induced cardiac miRNA transcriptome in healthy rats (ZL-Rap) are identical to 80% of the diabetes-induced cardiac miRNA transcriptome (ZO-C), which might be responsible for the rapamycin-induced insulin resistance. Using *in silico* analyses, the authors presented the interactions between differentially expressed cardiac cytokines and miRNAs, which might reflect both diabetes- and rapamycin-induced immune suppression. Several differentially expressed miRNA transcriptomes also serve as an adaptive mechanism to regulate cardiac fibrosis. This study provides a new insight for developing novel drugs, which can ameliorate the adverse effects of long-term treatment with rapamycin.

H. Merino and D. K. Singla reported the molecular mechanism underlying doxorubicin-induced apoptosis in soleus muscle using C57BL/6 mice. Their results suggest that doxorubicin treatment increases oxidative stress and apoptosis. Notably, it decreases antioxidants and antiapoptotic proteins, which are mediated through the Akt-mTOR pathway. Interestingly, tail vein injections of secreted frizzled-related protein-2 (sFRP2) blunt the detrimental effects of doxorubicin. Accordingly, they concluded that sFRP2 might be a valuable therapeutic candidate for doxorubicin-induced muscle toxicity.

Y. Huang et al. provided a mechanistic evidence of the beneficial effects of quercetin, a natural polyphenolic compound, in the neuronal tissue. They found that quercetin improves lysosome-mediated degradation and self-renewal in the neuronal tissue by inducing the nuclear translocation of transcription factor EB (TFEB). TFEB controls lysosome biogenesis, autophagy, and cellular trafficking in the phagocytic cells, like the retinal pigment epithelium (RPE). mTOR phosphorylates TFEB at its C-terminal serine-rich motif and thereby sequesters TEFB in the cytoplasm. Quercetin directly inhibits mTORC1 activation possibly by acting as a competitive mTOR kinase inhibitor at the ATP-binding motif; however, it does not influence the activity of Akt.

B. Huang et al. evaluated the relevance of gankyrin, a molecular chaperone that acts on assembly of 26S proteasome, specifically the 19S regulatory complex, in gastric cancer, a malignant epithelial tumor usually asymptomatic until late diagnosis associated with poor overall survival. Using samples of malignant infiltrating gastric cancer tissues and paired noncancerous tissues obtained from patients, as well as two gastric cancer cell lines, they suggested that gankyrin drives early malignant transformation of gastric cancer and alleviates oxidative stress via mTORC1 activation. The authors suggested that increased gankyrin expression could be a biomarker for early diagnosis of gastric cancer, which could be the risk factor of gastric cancer in patients with precancerous lesions such as dysplasia and intestinal metaplasia.

M. A. Ortega et al. performed an observational, analytical, and prospective cohort study on young (less than 50 years) and aged (more than 50 years) patients with and without valvular incompetence (venous reflux, which leads to chronic venous insufficiency, CVI). Their study was focused on the PI3K/Akt/mTOR pathway and inflammatory process by measuring the levels of CD4+, CD8+, and CD19+ cells. They also measured the levels of hypoxia-inducible factor-1*α* (HIF-1*α*) and HIF-2*α* expressions, which are induced in the heart deprived of oxygen supply. Their results showed an increased activity of the PI3K/Akt/mTOR pathway and upregulation of HIF-1*α*, CD4+, and CD8+ in young patients with valvular incompetence. It suggests that the PI3K/Akt/mTOR pathway may have an important role in CVI development in young patients.

W. Yu et al. determined the effect of exendin-4 and liraglutide, two glucagon-like peptide-1 (GLP-1) agonists, on glucose toxicity-induced cardiac injury through mTOR/ULK1-dependent autophagy. They treated primary cardiomyocytes from adult mice and H9C2 cardiomyocytes with high or normal dose of glucose with or without exendin-4 or liraglutide. They found that high-glucose treatment decreased cardiomyocyte contractility, which was partly restored by GLP-1 agonist treatment. GLP-1 agonist also rescued cardiomyocytes from glucose toxicity by inducing autophagy.

Mitofusin 2 (Mfn2), an outer mitochondrial membrane GTPase, is critical for mitochondrial fusion, which controls mitochondrial dynamics, distribution, and function within the cell. Intriguingly, emerging evidences identify the key role of Mfn2 in the onset/progression of different pathological conditions, including cancer. In this special issue, R. Xue et al. demonstrated that the overexpression of Mfn2 in pancreatic cancer cells inhibits proliferation and ROS generation, while inducing apoptosis. Mfn2 induces cellular autophagy of pancreatic cancer cells possibly by inhibiting the PI3K/Akt/mTOR signaling pathway. Authors found that pancreatic cancer patients with Mfn2-positive expression have significantly longer survival time than those with Mfn2-negative expression. Based on the bioinformatics analysis, they suggested that Mfn2 might be a potential therapeutic target in pancreatic cancer.

In the minireview article, A. Kezic et al. briefly summarized the metabolic adverse side effects (hyperglycemia, insulin resistance, and dyslipidemia) of chronic treatment with mTOR inhibitors (like macrolide rapamycin or other rapalogs), especially in patients with organ transplantation or cancer. The chronic pharmacological inhibition of activated mTOR may deteriorate the systemic metabolism in diabetes mellitus due to the pleiotropic effects of mTOR. Acute treatment with rapamycin or rapalogs specifically inhibits mTORC1 activity, without interfering the mTORC2 activity. However, a prolonged exposure of rapamycin or rapalogs leads to the suppression of mTORC2/Akt signaling, with consequent insulin resistance and insufficient immunosuppression. The authors compared the metabolic consequences of the chronic treatment with mTOR inhibitors with the metabolic profile provoked by metformin, a widely prescribed antidiabetes drug. Based on the literature, the authors proposed to use rapamycin/rapalogs in combination with metformin to induce AMPK activity, which might be a better therapeutic intervention to reduce the dose of rapamycin/rapalogs as well as associated adverse metabolic effects after solid organ transplantations.

A. Samidurai et al. comprehended our recent knowledge in the mechanisms of interactions between the mTOR signaling pathway and miRNAs (a class of short noncoding RNA) in cardiovascular diseases, like myocardial infarction, vascular remodeling and hypertrophy, heart failure, arrhythmia, and atherosclerosis. The authors also summarized the critical roles of miRNAs in the regulation of mTOR signaling in cardiovascular disease-associated risk factors, including diabetes and obesity. The review highlighted the latest advances on mTOR-targeted therapy and interactions of mTOR with miRNAs in clinical trials, which encourages us in exploring the novel therapeutics for heart disease with a unique perspective. Advancing our knowledge in the interplay between mTORC1 and mTORC2 complexes and its association with miRNAs could lead to the development of an efficient miRNA-based therapeutics and diagnostics for cardiovascular diseases.

S. D. Viana et al. focused their review article on the advances, drawbacks, and challenges regarding the use of mTOR inhibitors in four major classes of renal interventions/diseases: (1) kidney transplantation, (2) polycystic kidney diseases, (3) renal carcinomas, and (4) diabetic nephropathy. In this comprehensive review, the authors briefly revisited the mTOR components and signaling pathways and then addressed the pharmacological armamentarium targeting the mTOR pathway currently available in research and development stages, covering different generations of mTOR inhibitors and complementary approaches (allosteric mTOR inhibitors: rapamycin/rapalogs; dual PI3K/mTOR inhibitors; ATP-competitive inhibitors: mTOR kinase inhibitors; and new-generation drugs, namely, RapaLink-1). After a concise revision on the physiological role of mTOR in the kidney, S. D. Viana et al. critically reviewed the therapeutic use of mTOR inhibitors in the aforementioned renal conditions, using a translational perspective from preclinical data to current clinical applications. The authors concluded that although mTOR inhibitors (specifically rapamycin and everolimus) have been successfully used as immunosuppressive therapy for the prevention of allograft rejection, namely, in renal transplantation, further preclinical (and particularly clinical) data are still needed to understand the putative benefits of mTOR inhibitors against polycystic kidney diseases, renal carcinomas, and diabetic nephropathy.

D. Agostini et al. present a review article with the updated discoveries regarding the role of exercise in inhibiting the mTOR pathway in triple-negative breast cancer (TNBC), which is an aggressive carcinoma and has poor response to available chemotherapies. TNBC is associated with early recurrences. The authors focused on the biological mechanisms putatively involved in TNBC, including microRNAs. They also discussed the benefits evoked by distinct exercise and training protocols as well as nutrients on mTOR signaling that could be involved in TNBC initiation and progression. They suggested that exercise could ameliorate the TNBC risk and reduce the tumor burden by inhibiting PI3K-Akt-mTOR signaling, when canonical radio-, chemotherapies or chemical mTOR inhibitors are largely ineffective to prevent and manage the TNBC. In this sense, prescription and implementation of active lifestyles, including exercise/training and healthy nutritional habits, could have wide-ranging implications for society, which might improve conventional cancer treatment, including emotional and social wellbeing, in TNBC patients.

In conclusion, we believe that our series of special issues on this research topic published several new findings, which advanced our knowledge of the pivotal roles of mTOR signaling in developing an effective and safe therapeutic strategy for the growing prevalence of multiple pathological disorders.

## Figures and Tables

**Figure 1:**
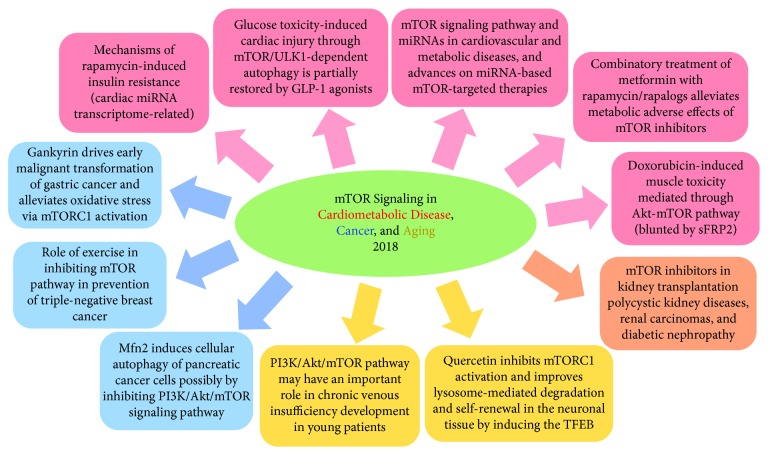
Key contents of all articles in the special issue on “mTOR Signaling in Cardiometabolic Disease, Cancer, and Aging 2018.”

